# Supplementary material for: Complex multiple introductions drive fall armyworm invasions into Asia and Australia
Source: Sci Rep. 2023 Jan 12;13:660. doi: 10.1038/s41598-023-27501-x (PMC9837037; doi:10.1038/s41598-023-27501-x)

**Complex multiple introductions drive fall armyworm invasions into Asia and Australia**

Rahul Rane^1†^, Thomas K Walsh^2†^, Pauline Lenancker^3^, Andrew Gock^2^, Thi Hang Dao^4^, Van Liem Nguyen^4^, Thein Nyunt Khin^5^, Divina Amalin^6^, Khonesavanh Chittarath^7^, Muhammad Faheem^8^, Sivapragasam Annamalai^8^, Sathis Sri Thanarajoo^8^, Y. Andi Trisyono^9^, Sathya Khay^10^, Juil Kim^11^, Lastus Kuniata^12^, Kevin Powell^3^, Andrew Kalyebi^13^, Michael H Otim^14^, Kiwoong Nam^15^, Emmanuelle d’Alençon^15^, Karl H. J. Gordon^2^, Wee Tek Tay^2†^

1. CSIRO, 343 Royal Parade, Parkville, VIC 3052, Australia

2. CSIRO, Black Mountain Laboratories, Clunies Ross Street, ACT 2601, Australia

3. Sugar Research Australia, 71378 Bruce Highway, Gordonvale, QLD 4865, Australia

4. Plant Protection Research Institute, Vietnam

5. Department of Agricultural Research, Myanmar

6. Department of Biology, De La Salle University, Philippines

7. Plant Protection Center, Department of Agriculture, Lao PDR

8. CAB International Southeast Asia, Malaysia

9. Department of Plant Protection, Faculty of Agriculture, Universitas Gadjah Mada, Indonesia

10. Plant Protection Division of CARDI, Ministry of Agriculture, Forestry and Fisheries, Cambodia

11. College of Agriculture and Life Science, Kangwon National University, Republic of Korea

12. New Britain Palm Oil, Ramu Agri Industry Ltd., Papua New Guinea

13. AJSK, P.O Box 16761, Kampala, Uganda.

14. National Crops Resources Research Institute, Namulonge, Kampala, Uganda

15. DGIMI, Université Montpellier, INRAE, Montpellier, France

† Applied BioSciences, Macquarie University, NSW, Australia

**Correspondence:** weetek.tay@csiro.au

**Supplemental Figures**

**Figure S1-S6:** DivMigrate analysis of invasive *Spodoptera frugiperda* populations from Africa (Benin (BEN), Uganda (UGA), Malawi (MWI)), South Asia (India (IND), East Asia (China (CHN), South Korea (KOR)), Southeast Asia (Myanmar (MMR), Laos People Democratic Republic (LAO), Viet Nam (VNM), Philippines (PHL), Malaysia (MYS)), and Papua New Guinea (PNG). Multiple populations from the Yunnan province in China (Cangyuang (CY), Yuanjiang (YJ), Xinping (XP)) and from the Penang (PN) and Johore (JB) states in Malaysia were included. Estimates of migration rates were calculated using G_ST_ with significant rates (shown as red values) at alpha = 0.5 estimated from 100 bootstrap replications. Note that no migration events were established for Benin and South Korea, suggesting unique introduction events among the invasive populations included in this analysis. Australian and Malaysia Kedah state populations were excluded to enable ease of interpreting migration patterns. See main text for detailed discussion.


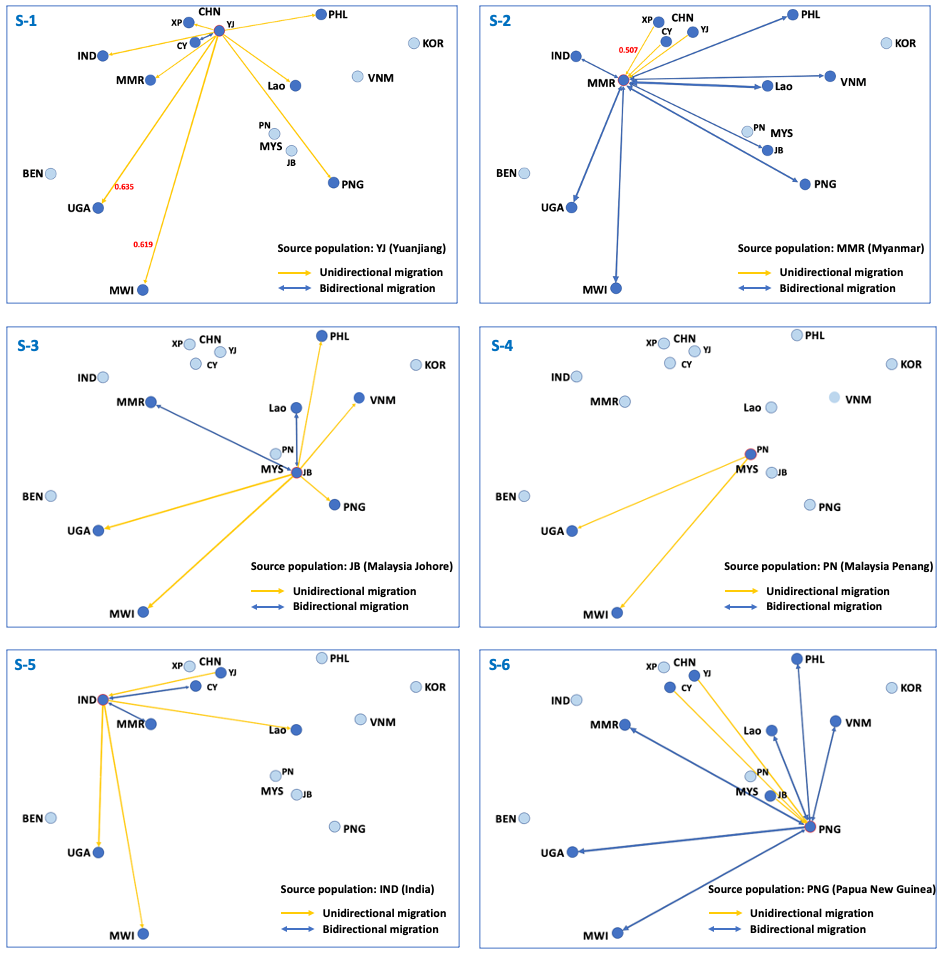

Supplement: Supplementary file 1 — Supplementary Figures. [file 41598_2023_27501_MOESM1_ESM.docx]
